# Detection of αB-Conotoxin VxXXIVA (αB-CTX) by ic-ELISA Based on an Epitope-Specific Monoclonal Antibody

**DOI:** 10.3390/toxins14030166

**Published:** 2022-02-23

**Authors:** Hengkun Tang, Haimei Liu, Yehong Gao, Rui Chen, Mingke Dong, Sumei Ling, Rongzhi Wang, Shihua Wang

**Affiliations:** Key Laboratory of Pathogenic Fungi and Mycotoxins of Fujian Province, Key Laboratory of Biopesticide and Chemical Biology of Education Ministry, School of Life Sciences, Fujian Agriculture and Forestry University, Fuzhou 350002, China; tanghengkun@fafu.edu.cn (H.T.); wuhuijuan@fafu.edu.cn (H.L.); gaoyehong@fafu.edu.cn (Y.G.); chenrui@fafu.edu.cn (R.C.); dmkhubei@fafu.edu.cn (M.D.); lsmpu2008@m.fafu.edu.cn (S.L.)

**Keywords:** αB-conotoxin VxXXIVA, epitope, hybridoma, monoclonal antibody, ELISA

## Abstract

In view of the toxicological hazard and important applications in analgesics and cancer chemotherapeutics of αB-CTX, it is urgent to develop an accurate, effective and feasible immunoassay for the determination and analysis of αB-CTX in real samples. In this study, MBP-αB-CTX4 tandem fusion protein was used as an immunogen to elicit a strong immune response, and a hybridoma cell 5E4 secreting IgG2b against αB-CTX was successfully screened by hybridoma technology. The affinity of the purified 5E4 monoclonal antibody (mAb) was 1.02 × 10^8^ L/mol, which showed high affinity and specificity to αB-CTX. Epitope 1 of αB-CTX is the major binding region for 5E4 mAb recongnization, and two amino acid residues (14L and 15F) in αB-CTX were critical sites for the interaction between αB-CTX and 5E4 mAb. Indirect competitive ELISA (ic-ELISA) based on 5E4 mAb was developed to detect and analyze αB-CTX in real samples, and the linear range of ic-ELISA to αB-CTX was 117–3798 ng/mL, with a limit of detection (LOD) of 81 ng/mL. All the above results indicated that the developed ic-ELISA had high accuracy and repeatability, and it could be applied for αB-CTX detection and drug analysis in real samples.

## 1. Introduction

Recently, cone snails have been regarded as one of the focuses for new drug discovery [1], with the majority of their distribution throughout tropical and subtropical waters, such as the South China Sea, Australia, and the Pacific Ocean [1,2]. Conotoxins, a diverse array of unique bioactive neurotoxins, are mainly secreted by different kinds of cone snails [3,4]. Conotoxins are often toxic to humans and animals and can cause convulsions and paralysis, even to death [5]. More than 80,000 natural conotoxins have been estimated to exist in various cone snails around the world [6,7,8], and all conotoxins can be divided into 26 gene superfamilies based on their conserved signal sequences and their characteristic cysteine framework [5,9]. Among them, α-, µ- and ω-conotoxins are the most characterized families so far for their high specificity and affinity to ion channels [1,10,11,12]. 

αB-conotoxin VxXXIVA (αB-CTX) is a new conotoxin found in *Conus vexillum*, which belongs to the B-gene superfamily. It is reported that the precursor of αB-CTX is different from other conotoxins as it lacks a pro region responsible for enhancing oxidative folding and secretion of hydrophobic O-superfamily conotoxins [12]. Specifically, such a mechanism is not necessary for the more hydrophilic αB-CTX. The mature αB-CTX is a peptide with 40 amino acid residues containing only 4 cysteine residues, and the most active disulphide linkages of αB-CTX are C-CC-C [12]. αB-CTX is a specific nicotinic acetylcholine receptor (nAChR) antagonist with the greatest potency against the α9α10 subtype [12]. The α9α10 nAChR is an important target for the development of analgesics and cancer chemotherapeutics [13,14,15,16], and αB-CTX represents a novel ligand with which to probe the structure and function of this protein. Given the toxicological effects and potential application value of αB-CTX, it is urgent to develop an accurate and sensitive method to identify and detect αB-CTX in sea samples.

At present, the main analysis and detection methods for αB-CTX are analytical chemistry, including high-performance liquid chromatography (HPLC), nuclear magnetic resonance (NMR) and circular dichroism (CD) [12]. Although these methods have high sensitivity and accuracy for analysis of the content and composition of αB-CTX, the main disadvantages of these methods are that they are time-consuming, require complex sample pre-treatment and require expensive equipment and trained professionals [17,18]. Moreover, complicated and tedious operation steps, high requirement of samples and detection practicability seriously limits their application in the detection of αB-CTX in real samples [19]. In contrast, immunoassays with high sensitivity, accuracy and adaptability have been widely used to detect the targets (toxins, pathogenic molecules and heavy metals) in different samples [20,21,22]. In the previous study, we developed ELISA and colloidal test strips based on monoclonal antibodies (mAb) against ω-CTX to detect ω-CTX MVIIA residue in Conus samples [5]. Specifically, no related immunoassay was reported to detect and analyze αB-CTX in real samples until now. This is may be due to the difficulty in acquiring antigens or the low immunogenicity of antigens, which fail to induce a strong immune response for antibody production. Fortunately, the antigen epitope analysis and antigen tandem fusion expression established in this study can effectively solve these key problems and can be used to solve these important limits. Therefore, the aim of this study was to design and prepare an antigen with strong immunogenicity to elicit the antibody production in mouse models and to screen an epitope-specific monoclonal antibody by hybridoma technology. The key binding regions and sites between the resulted mAb and αB-CTX were further analyzed. Finally, ic-ELISA based on epitope-specific mAb was established and used to detect and analyze the content of αB-CTX toxin in actual samples.

## 2. Results and Discussion

### 2.1. Epitope Binding Pattern Evaluation

**Figure 1:**
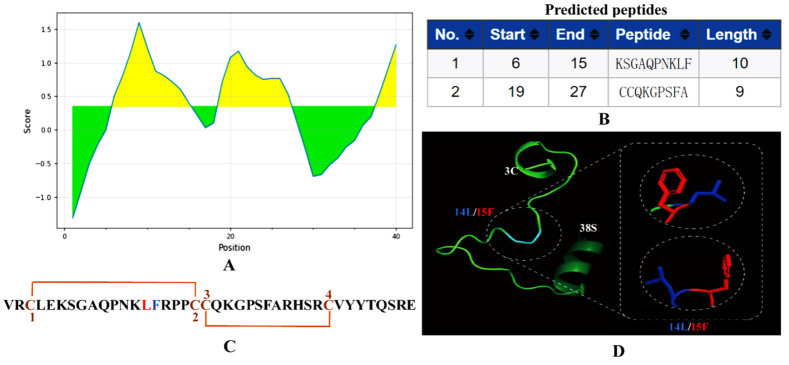
Antigen epitope prediction and 3D structure analysis. (**A**) Output graph of the predicted B-cell linear epitopes showing the amino acid position (x-axis) and Bepipred score (Y-axis). Residues (yellow) with scores above the default threshold of 0.35 have a higher probability of being part of an epitope. The green indicates lower probability of being part of an epitope. (**B**) Two predicted epitopes of lengths (start and end positions were indicated; 1: EP1 (6–15); 2: EP2: (19–27)). (**C**) Sequence and disulfide connectivity of αB-CTX. (**D**) Swiss model of αB-CTX from Cys3 to Ser38 using Leishmania Major mitochondrial ribosome (PDB entry 7ane.36.A) as template. Amino acid residues Leu14 and Phe15 are shown in dark blue and red, respectively. The sequences upstream of Cys3 and downstream of Ser38 were not modeled.

αB-CTX is a peptide consisting of 40 amino acid residues in length. The antigenic sites on αB-CTX were first analyzed using the Bepipred B-cell linear epitope prediction tool [23]. As shown in Figure 1, this antigen contains two major epitopes located at positions 6–15 and 19–27 in the protein sequence, and the regions of the predicted antigenic sequences were designated as EP1 and EP2, respectively (Figure 1A,B). Interestingly, the αB-CTX has four Cys residues on its peptide sequence, and the specific disulfide bond arrangement of this peptide is C-CC-C (Figure 1C, chocolate yellow) [12]. Meanwhile, the predicted 3D model of αB-CTX resembles a triangular clamp, with a helix at each end. The key binding sites (Leu14 and Phe15) between antibody and antigen interactions were marked as different colors in the predicted 3D model and linear amino acid sequences (Figure 1D, dark blue and red).

### 2.2. Preparation of αB-CTX and Animal Immunization

**Figure 2:**
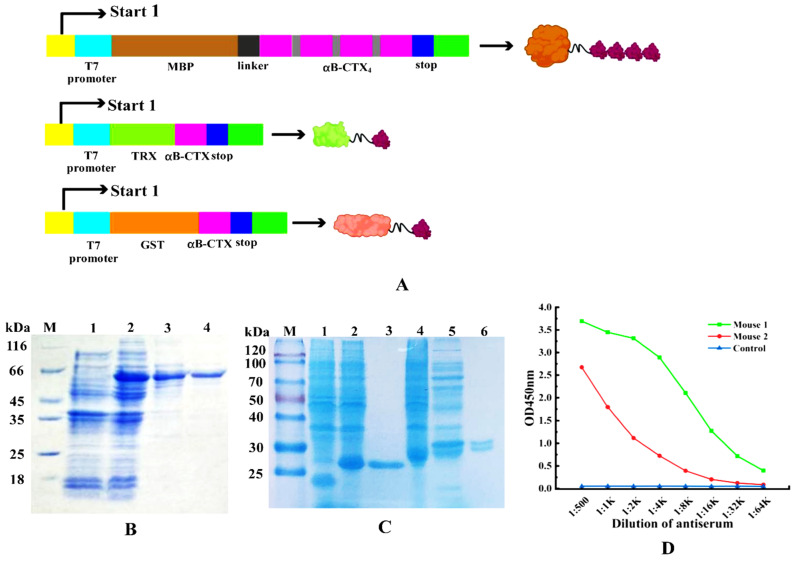
Purification of fusion proteins and animal immunization. (**A**) Construction of fusion protein expression vectors (MBP-LK-αB-CTX4, TRX-αB-CTX and GST-αB-CTX). (**B**) Expression and purification of fusion proteins MBP-LK-αB-CTX4. Lane M: middle molecular protein marker, Lane 1: the expressed product of pBD-*mbp*/BL21(DE3), Lane 2: the expressed total proteins of pBD-*mbp-lk-αB-CTX4*/BL21(DE3), Lane 3–4: the purified product of MBP-LK-αB-CTX4. (**C**) Expression and purification of fusion proteins TRX-αB-CTX and GST-αB-CTX. Lane M: middle molecular protein marker, Lane 1: the expressed product of pET-32a/BL21(DE3), Lane 2: the expressed total proteins of pET-32a-*αB-CTX*/BL21(DE3), Lane 3: the purified product of TRX-αB-CTX, Lane 4: the expressed product of pGEX-6P-1/BL21(DE3), Lane 5: total proteins of pGEX-6P-1-*αB-CTX*/BL21(DE3), Lane 6: the purified product of GST-αB-CTX. (**D**) Titer determination of the immunized mouse by ELISA.

The connected tetramer fragments (αB-CTX4) and αB-CTX monomer were used to construct three different expression vectors for protein expression and purification (Figure 2A). The fusion protein MBP-LK-αB-CTX4 and the two other fusions (TRX-αB-CTX and GST-αB-CTX) were shown in Figure 2B,C. These fusion proteins were successfully expressed after IPTG induction and further purified by Ni^2+^-NTA affinity chromatography. The band sizes of the purified MBP-LK-αB-CTX4 and TRX-αB-CTX with high purity on the gel of SDS-PAGE were 60 kDa and 28 kDa, respectively, the same as the theoretical result [5]. Then, the purified MBP-LK-αB-CTX4 was used as the immunogen for animal immunization, and the titer of serum was tested by ELISA with the TRX-αB-CTX as the detection antigen (Figure 2D). Compared to the immunized mouse 2 and control, the immunized mouse 1 had the highest antiserum titer to the target antigen (reaching 1:16,000), indicating that a strong immune response was induced by the MBP-LK-αB-CTX4 antigen.

### 2.3. Screening and Identification of Positive Hybridoma

**Figure 3:**
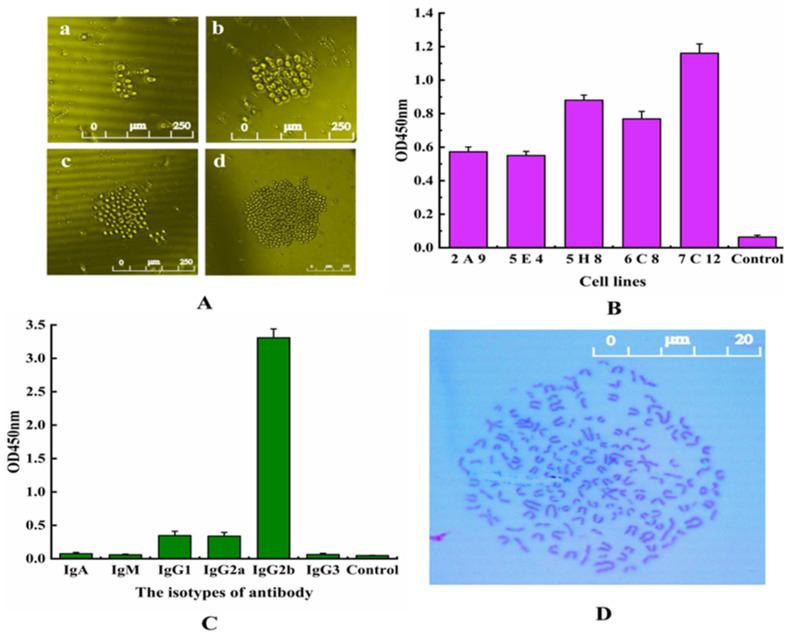
Hybridoma screening and characterization. (**A**) Hybridoma cell culture after fusion. a: the 3rd day, b: the 5th day, c: the 7th day, d: the 9th day. (**B**) ELISA assay of the selected positive hybridoma cell lines. Five positive hybridoma cell lines were obtained and named 2E9, 5E4, 5H8, 6C8 and 7C12. (**C**) Isotyping of 5E4 cells secreting anti-αB-CTX mAb by using an isotyping kit. (**D**) Analysis of the chromosome of 5E4 cell line.

After fusion, the cells in the 96-well plate were composed of 3 kinds of cells: myeloma cells, mouse spleen cells and feeder layer cells. These cells were cultured in a selective medium containing 2% HAT and 20% FBS [20]. From the third day, the fusion cell colonies could be obviously observed and gradually grown as time went on, and most of the unfused cells were apoptotic (Figure 3A). On the 8th day after fusion, the supernatant of the culture was taken to detect whether the cells secreted antibodies by ELISA, and 5 positive hybridomas were screened out successfully. Combined with the growth status of cells and antibody properties, the hybridoma 5E4 was finally selected for subsequent experiments (Figure 3B). As shown in Figure 3C, the IgG2b subtype has the highest value, while the values of other types are very low, further indicating that the subtype of the 5E4 cell line belongs to IgG2b. The chromosome analysis result of hybridoma 5E4 in Figure 3D showed that the chromosome numbers of hybridoma 5E4 were 106 ± 2, corresponding to the sum of the chromosome number of myeloma cells and mouse spleen cells in theory [21]. The ideal hybridoma should be selected based on multi-factors. Although the supernatant titer of hybridoma 5E4 was not the best in Figure 3B, the antibody secreted by 5E4 exhibited the best specificity and stability to αB-CTX compared to other hybridomas. Hence, it was chosen as the ideal cell line for further antibody purification.

### 2.4. Purification and Identification of 5E4 mAb

**Figure 4:**
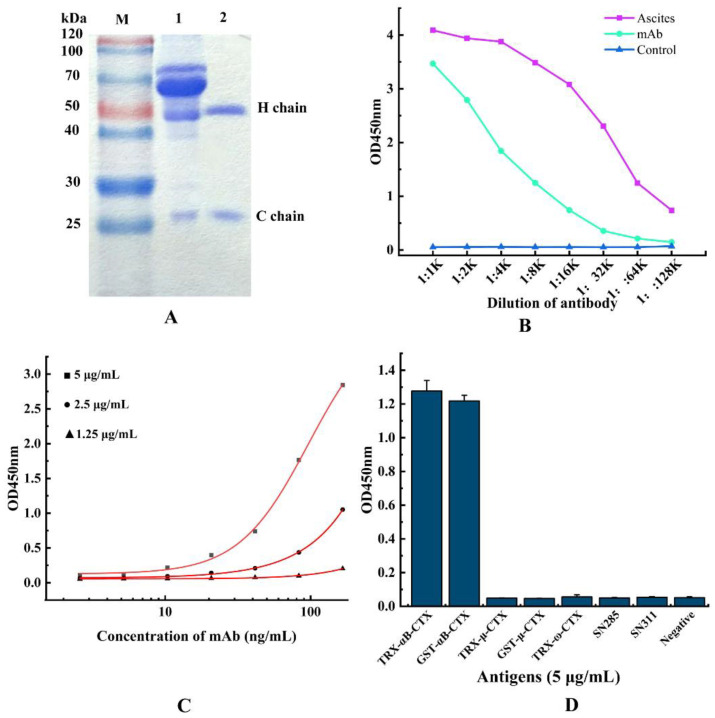
Purification and characterization of anti-αB-CTX mAb. (**A**) SDS-PAGE analysis of the purified mAb. Lane M: standard protein marker, Lane 1: the total protein of ascites, Lane 2: the purified anti-αB-CTX mAb. (**B**) The titer of ascites and anti-αB-CTX mAb. (**C**) Affinity determination of anti-αB-CTX mAb by iELISA. The affinity constant of the αB-CTX mAb is 1.02 × 10^8^ L/mol. (**D**) The specificity of anti-αB-CTX mAb was determined by ic-ELISA.

To obtain the monoclonal antibody 5E4 with high affinity and purity, the cultured hybridoma 5E4 cells were injected into the abdominal cavity of pre-sensitized mice to produce ascites, and the anti-αB-CTX4 mAb was purified by Protein G affinity chromatography. SDS-PAGE results, as shown in Figure 4A, indicated that the purified antibody has two protein bands with molecular weights of 50 kDa and 25 kDa on gel, corresponding to the heavy chain and light chain, respectively. The titer of ascites and purified antibody were shown in Figure 4B, and the purified 5E4 mAb exhibited similar αB-CTX binding activity to that of ascites. In this experiment, the affinity of the resulting 5E4 mAb was determined by indirect ELISA (iELISA), and the affinity curve was drawn using Origin 8.0 software. The results, shown in Figure 4C demonstrated that the affinity of 5E4 mAb was 1.02 × 10^8^ L/mol, belonging to high affinity antibody. Meanwhile, the specificity of 5E4 mAb was further analyzed, and the result was shown in Figure 4D. As expected, the prepared 5E4 mAb could specifically bind to the two different fusion proteins GST-αB-CTX and TRX-αB-CTX in ELISA assay, but not react with the other antigens, such as GST-μ-CTX, TRX-μ-CTX, TRX-ω-CTX, SN311 and SN285, indicating that this 5E4 mAb with high affinity was specific to αB-CTX.

### 2.5. αB-CTX (1–20) Is the Major Binding Region by 5E4 mAb Recognition

**Figure 5:**
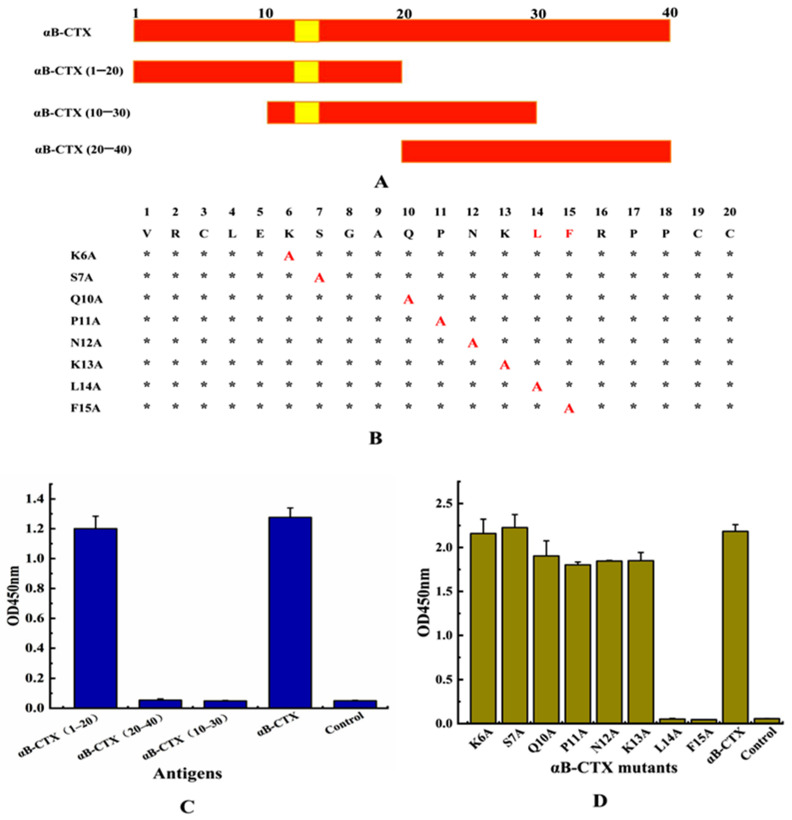
Epitope identification for 5E4 anti-αB-CTX mAb interactions. (**A**) Three regions of αB-CTX, including potential binding sites (Val1-Cys20, Gln10-Ser30 and Cys20-Glu40). (**B**) Epitope mapping of region Val1-Cys20 by alanine-scan mutagenesis of the mAb binding site. Critical residues (highlighted in red) are defined as those with alanine substitution. *: the unmutated amino acid site; A: the amino acid was mutated to alanine. (**C**) The titer for various peptide regions and αB-CTX full-length peptide. (**D**) The titer for various site mutants and αB-CTX full-length peptide.

After the preparation of the antibody, how to investigate the binding area for 5E4 mAb-αB-CTX interactions became one of the main concerns of this study. To achieve this purpose, full-length αB-CTX and three different αB-CTX fragments including αB-CTX (1–20), αB-CTX (10–30) and αB-CTX (20–40) were designed and synthesized (Figure 5A), and ELISA was used to test the binding activity of these fragments. The ELISA results in Figure 5C showed that the purified 5E4 mAb has a strong binding signal to αB-CTX (1–20), similar to that of full-length αB-CTX, with the binding activity being far higher than others. Compared to αB-CTX (1–20), αB-CTX (10–30) and αB-CTX (20–40) are not recognized by the 5E4 mAb. The result derived from the ELISA indicated that αB-CTX (1–20) is the major binding region for 5E4 mAb recognition. Interestingly, the predicted first epitope 1 (EP1) of αB-CTX was just located in the region of 6–15 (Figure 1B), further suggesting that the EP1 may be the major region for 5E4 mAb recognition.

### 2.6. L14 and F15 Are the Critical Sites between 5E4 mAb-αB-CTX Interactions

To further analyze the critical sites responsible for 5E4 mAb-αB-CTX interactions, 8 amino acids located at the epitope 1 (6–15) of αB-CTX, except for G8 and A9, were mutated to alanine by site-directed mutagenesis (Figure 5B). The ELISA result in Figure 5D showed that the binding activity of 5E4 mAb against 2 mutated amino acids (L14A and F15A) was all obviously decreased, similar to the negative control. Other mutants (K6A, S7A, Q10A, P11A, N12A and K13A) exhibited strong binding signals to 5E4 mAb recognition as the maternal αB-CTX. The above results demonstrated that amino acids L14 and F15 located at the epitope 1 of αB-CTX were critical sites for the interaction between 5E4 mAb and αB-CTX.

### 2.7. Establishment of ic-ELISA for αB-CTX

**Figure 6:**
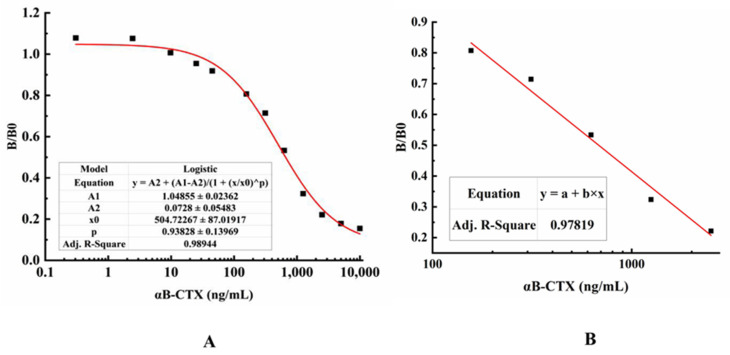
Standard curves for αB-CTX detection. (**A**) A typical calibration curve illustrated by plotting (B/B0) against αB-CTX. The data obtained with various inhibitor concentrations and without inhibitor are referred to as B and B0, respectively. The equation is y = 0.0728 + (1.04855 − 0.0728)/((1 + (x/504.72267)^0.93828^), with a correlation coefficient (R^2^) of 0.98944. The limit of detection (LOD) was 81 ng/mL for αB-CTX. (**B**) The linear portion of the standard curve. The equation is y = 1.97071 − 0.51887x, with a correlation coefficient (R^2^) of 0.97819. The range of linear detection for αB-CTX was 117–3798 ng/mL.

αB-CTX is a hapten with low molecular weight and limited epitopes, so it is very difficult to develop a double antibody sandwich ELISA for αB-CTX detection and analysis. Combining the situation of 5E4 mAb and αB-CTX, indirect competitive ELISA (ic-ELISA) was applied to detect αB-CTX in real samples after optimizing the antigen and antibody concentration. As shown in Figure 6, the typical calibration curve was drawn by plotting (B/B0) against αB-CTX concentration, and the equation of the logistic curve was y = 0.0728 + [(1.04855 − 0.0728)/(1 + x/504.72267)^0.93828^], with a correlation coefficient (R^2^) of 0.98944 (Figure 6A). From the result shown in Figure 6B, the linear equation was y = 1.97071 − 0.51887x, and the correlation coefficient (R^2^) was about 0.97819. In ic-ELISA, the limit of detection (LOD) for αB-CTX was 81 ng/mL, which is defined as the concentration of target antigen corresponding to the inhibition rate that reached 10% [19,24]. Furthermore, the linear range of detection was 117~3798 ng/mL with a half inhibitory concentration (IC50) of 661 ng/mL.

### 2.8. Detection of αB-CTX by ic-ELISA in Spiked and Real Samples

In this study, the recovery and variation coefficients for αB-CTX detection were calculated by ic-ELISA, and the PBSM solution without any contamination was spiked with different concentrations of αB-CTX [18]. All analyses were performed with three parallel experiments. As shown in Table 1, the recovery of detection ranged from (86.62 ± 0.72)% to (96.46 ± 6.09)%, with an average of (92.93 ± 3.69)%, and the average variation coefficient (CV) was 3.87% in intra-assay. The average recovery and CV in inter-assay were (96.31 ± 4.48)% and 4.52%, respectively. The results from ELISA detection indicated that the developed ic-ELISA had high accuracy and repeatability, and it could be used to detect the content of αB-CTX in real samples. Finally, five different shellfish seafood products were purchased randomly from local supermarkets as experimental detection materials by ic-ELISA. The detection results in Table 2 showed that the values of these real samples were consistent with that of PBS, indicating that none of these actual samples contained α-CTX toxin.

## 3. Discussion

The previous study in our laboratory has shown that the monomer αB-CTX cannot induce the production of an antiserum with high binding activity, failing to stimulate a strong immune effect (data not shown). When smaller antigens, especially the ploy-peptides with low molecular weights, were used, the immunogenicity was often limited or invalid [5]. To effectively solve this critical problem, the four original genes of αB-CTX were connected in series to form tetramers in this study, and the purified MBP-LK-αB-CTX4 fusion protein was used as an immunogen to elicit a strong response for antibody production. Compared to the control, the titer of serum from mice immunized by MBP-LK-αB-CTX4 was very high. In theory, the polymerization state of the antigen is an important factor affecting the immunogenicity, and the immunogenicity of the polymer is usually stronger than that of the monomer. The fusion protein MBP-LK-αB-CTX4 containing tetramer form has a larger molecular weight and better protein folding, which is easier to stimulate the immune effect of the body. As expected, the ELISA result from the antiserum titer also strongly confirmed this judgment. Therefore, the method used in this study by connecting antigens with a linker to form a multimer is feasible for improving the immunogenicity of the antigen, and it may provide a useful idea for solving similar critical problems in polypeptide antigens.

In the process of fusion, it should be noted that the spleen and connective tissue should be stripped clearly in a clean environment, and the optimal ratio of spleen cells and myeloma cells was controlled at 3:1~5:1. The addition of feeder layer cells is necessary for cell fusion, and the growth-promoting factors and nutrients secreted by the feeding layer of cells provide a comfortable and favorable environment for the growth of hybridomas [24]. How to maintain high activity of antibody has been a topic of constant concern. According to our experience, low temperature (0 °C~4 °C) and a solution with an accurate pH value (pH 7.2) are two necessary conditions to ensure the activity of antibody in the process of antibody purification.

To ensure the accurancy of ic-ELISA, it is necessary that the concentration of antigen and antibody should be accurately measured during the experiments by using multiple checkerboard iELISA experiments, and the dilution should be as accurate as possible. In fact, the accuracy of ic-ELISA can be affected by many factors, including the coating time, the choice of coating and block buffer, and the concentration of substrate and antibody [5]. To improve the accuracy of the ic-ELISA detection, the above factors should be maintained at the same condition throughout the system when the optimal conditions and reagents are explored clearly. The best coating time in this study is at 4 °C overnight, and the optimal dilution ratio of HPR-goat anti-mouse IgG antibody and antigen coating concentrations were 1:16,000 and 10 μg/mL, respectively.

## 4. Conclusions

In the present study, an effective antigen tandem design and expression format was used to increase the immunogenicity of the target antigen, further solving the key problem of antibody preparation. A hybridoma 5E4 stably generating mAb against αB-CTX was prepared for the first time through hybridoma technology. The resulting 5E4 mAb was specific to epitope 1 of αB-CTX, and the amino acids L14 and F15 were two critical sites for 5E4 recognition. The affinity of the purified 5E4 mAb was 1.02 × 10^8^ L/mol, belonging to a high-affinity antibody. The developed ic-ELISA in this study based on 5E4 mAb has good specificity and accuracy, and the average recovery and CV were (92.93 ± 3.69)% and 3.87%, respectively. Under optimal conditions, the LOD of ic-ELISA was 81 ng/mL, with a linear range of 117–3798 ng/mL. Lastly, no αB-CTX was detected in five different actual shellfish seafood products. In conclusion, the developed ic-ELISA could be used as a useful and feasible method for the analysis and detection of αB-CTX in real samples.

## 5. Materials and Methods

### 5.1. Materials

Balb/c mice were purchased from Wushi Animal Laboratory (Shanghai, China). Hypoxanthine, aminopterin and thymidine supplement (HAT) was obtained from Sigma-Aldrich Chemical (St. Louis, MO, USA). BCA protein assay kit (CAT: PC0020) was purchased from Solarbio Life Science (Beijing, China). All other used chemicals were of analytical grade. All the animal experiments in the present study complied with the Research Ethics Committee of Fujian Agriculture and Forestry University, China (Permit No. PFMFAFU201610) [5,17].

### 5.2. Epitope Predication and 3D Structure Simulation

The mature peptide sequence and cysteine framework of αB-CTX were referred to as in the previous report [12], and the possible epitopes of αB-CTX were further analyzed using the B-Cell Linear Epitope Prediction tool on the Immune Epitope Database Analysis Resource server (http://tools.immuneepitope.org/bcell/) (accessed on 6 January 2022) [23,25]. Meanwhile, the 3D structure of αB-CTX was predicated on the basis of homology modeling using SWISS-MODEL (http://swiss.model.expasy.org/) (accessed on 6 January 2022) [26,27,28].

### 5.3. Protein Expression and Purification

To achieve high expression and purification of αB-CTX in *E. coli* BL21 (DE3), the DNA fragment encoding mature αB-CTX peptide was synthesized by Shanghai Biotech Co., Ltd. (Shanghai, China) after codon optimization [29]. The resulting DNA fragment was inserted into the plasmids pET32a and pBD-mbp for the construction of recombinant expression vectors, and the target proteins were expressed in *E. coli* BL21 (DE3) by IPTG induction (a final concentration of 1.0 mM) when the cultures reached an OD_600_ of 1.0 [30]. After cultivation, the fusion proteins were purified by Ni^2+^-NTA affinity chromatography, and the protein concentration was determined by SDS-PAGE and BCA methods [18].

### 5.4. Animal Immunization and Titer Determination

To effectively activate the immune response of the mouse model, the purified MBP-LK-αB-CTX4 fusion protein was used as the immunogen, and the procedures of animal immunization and titer determination were performed as described with minor modifications [17,31]. Balb/C mice at the age of 7 weeks were immunized subcutaneously (100 μg MBP-LK-αB-CTX4 emulsified in Freund’s complete adjuvant) with an interval of 2 weeks. A total of 5 days after the 5th immunization, the ELISA was used to test the titer of serum from the control and each immunized mouse. The mouse with the highest antibody titer was regarded as the spleen donor for cell fusion and hybridoma screening.

### 5.5. Screening and Identification of Positive Hybridoma

The detailed protocols of cell fusion and hybridoma selection were carried out as described in the referred methods [5,12]. The subclass of the antibody secreted by the positive hybridoma was identified by using Mouse Monoclonal Antibody Subtyping Kit [21], and the chromosome numbers of selected hybridoma cells were observed and counted using Jimsey staining solution as described [5].

### 5.6. Purification and Identification of mAb against αB-CTX

To obtain a monoclonal antibody (mAb) with high binding activity and specificity for further investigation, healthy Balb/C mice aged 8–12 weeks were sensitized by paraffin at the abdominal cavity, and then the cultured positive hybridoma (about 10^6^ cells) was injected into the abdominal cavity of the mice for ascites production. The caprylic/ammonium sulfate precipitation and protein A/G methods were used to purify the IgG mAb against αB-CTX from ascites fluid, and the concentration and fineness of purified antibody were analyzed by the SDS-PAGE and BCA kits [5]. The binding activity (affinity) and specificity of the purified anti-αB-CTX mAb were determined by indirect ELISA as described [19].

### 5.7. Analysis of Different Binding Epitopes

To investigate the binding epitope of the prepared mAb to αB-CTX, αB-CTX was divided into three different fragments according to the protein sequence, and the resulting fragments were designated as αB-CTX (1–20), αB-CTX (10–30) and αB-CTX (20–40), respectively. These three polypeptides were synthesized by Shanghai Biotech Co., Ltd. (Shanghai, China) and then characterized by HPLC. Furthermore, 3 different peptides and the full-length αB-CTX protein were used as the antigen to coat the 96-well plates (10 μg/mL), and the purified 5E4 mAb in PBSM solution (1:4000 dilution) was added to the reaction wells after blocking and washing. The binding validation was then performed by ELISA as described [32].

### 5.8. Analysis of Specific Sites between mAb and αB-CTX Interaction

In view of the research results of antibody binding to a specific epitope, the critical residues for mAb-αB-CTX binding were further determined. A total of 8 amino acids located on epitope 1 of αB-CTX, regarded as the specific binding epitope for antigen-antibody interactions, were individually mutated to alanine [32]. In the same way, the constructed αB-CTX mutants were synthesized by Shanghai Biotech Co., Ltd. (Shanghai, China) and then characterized by HPLC. Then, 8 mutated αB-CTX proteins and αB-CTX were used as the antigen to coat the 96-well plates (10 μg/mL), and the purified 5E4 mAb in PBSM solution (1:4000 dilution) was added to the reaction wells after blocking and washing. The binding activities of mAb to αB-CTX mutants were determined by ELISA according to the instructions described above.

### 5.9. Establishment of ic-ELISA and Standard Curve

To effectively detect and analyze the content of αB-CTX in samples, indirect competitive ELISA (ic-ELISA) was established via a specific antibody-antigen reaction [24]. For testing the best concentration on the calibration curve, the standard αB-CTX protein (as a complete antigen) was 2-fold serially diluted (10~1.25 μg/mL, 625~1.2125 ng/mL and 606~1.18 pg/mL, respectively) to react with the anti-αB-CTX mAb in a tube at 37 °C for 30 min. Then, the reaction mixtures were added into the reaction wells with three replicates of each concentration standard. The data from ic-ELISA was calculated and analyzed, and the typical calibration curve and linear portion of the standard curve were illustrated by using Origin 8.0 software according to the value of plotting (B/B0) against the αB-CTX concentration [24]. In ic-ELISA, B0 is the OD450 nm value of the control well without inhibitor, and B is the OD450 nm value of the well with inhibitor. B/B0 is called the inhibition rate. IC50 indicates the concentration of inhibitor when the inhibition rate reaches 50%.

### 5.10. αB-CTX Detection in Spiked and Real Samples by ic-ELISA

To further assess the accuracy and recovery of the developed ic-ELISA method, the PBSM (PBS containing 5% skim milk powder) solution (no αB-CTX protein) was treated with different concentrations of standard αB-CTX as spiked samples. To test and assess the recovery and accuracy of ic-ELISA detection, the inter-assay and intra-assay were performed with three replicates of each concentration standard, and the average recovery and coefficient of variation (CV) were defined as the ratio of standard deviation to the mean in recovery test [5]. At last, five different snail and shell samples purchased randomly from Fuzhou supermarkets were detected using the ic-ELISA developed in this study.

### 5.11. Statistical Analysis

All the experimental data were processed and analyzed by ORIGIN 8.0 (OriginLab, Northampton, MA, USA). Curve fitting and variance analysis were conducted to describe the relationship between variables. All analyses were performed with three parallel tests.

## Figures and Tables

**Table 1:** The determination of recovery from spiked samples.

Spiked Level (ng/mL)	Intra-Assay				Inter-Assay			
		Measured	Recovery	CV		Measured	Recovery	CV
	*n*	(ng/mL)	(%)	(%)	*n*	(ng/mL)	(%)	(%)
2500	3	2165.54 ± 18.03	86.62 ± 0.72	0.83	3	2190.13 ± 42.49	87.6 ± 1.7	1.94
600	3	578.74 ± 36.56	96.46 ± 6.09	6.31	3	597.1 ± 24.89	99.5 ± 4.15	4.17
150	3	143.55 ± 6.39	95.7 ± 4.26	4.45	3	152.71 ± 11.39	101.8 ± 7.59	7.46
Average	-	-	92.93 ± 3.69	3.87	-	-	96.31 ± 4.48	4.52

**Table 2:** The detection result of αB-CTX toxin in real samples.

Samples		OD450 nm Value	Detection Results
*Oncomelania hupensis Gredler*	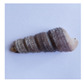	0.9983 ± 0.0366	-
Shellfish (qīng é)	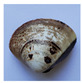	1.001 ± 0.0669	-
*Ruditapes philippinarum*	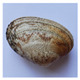	0.953 ± 0.0321	-
*Viviparidae*	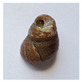	1.0143 ± 0.0598	-
*Thais clavigera Kuster*	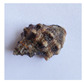	0.968 ± 0.0363	-
αB-CTX		0.0545 ± 0.0048	+
PBS		0.9805 ± 0.1158	-

Detection results of αB-CTX in real samples (*n* = 3). +: indicates that αB-CTX is detected in real samples. -: indicates that no αB-CTX is detected in real samples.

## Data Availability

Not applicable.
